# Clinical judgement and the medical profession

**DOI:** 10.1111/j.1365-2753.2010.01560.x

**Published:** 2011-08

**Authors:** Gunver S Kienle, Helmut Kiene

**Affiliations:** Senior Researcher, Institute for Applied Epistemology and Medical MethodologyFreiburg i.Br., Germany

**Keywords:** art and science of medicine, causality assessment, clinical judgement, clinical research, Gestalt cognition, medical epistemology, professionalism in medicine, tacit knowledge

## Abstract

**Objectives:**

Clinical judgment is a central element of the medical profession, essential for the performance of the doctor, and potentially generating information also for other clinicians and for scientists and health care managers. The recently renewed interest in clinical judgement is primarily engaged with its role in communication, diagnosis and decision making. Beyond this issue, the present article highlights the interrelations between clinical judgement, therapy assessment and medical professionalism.

**Methods:**

Literature review and theory development.

**Results:**

The article presents different methodological *approaches to causality assessment* in clinical studies and in clinical judgement, and offers criteria for clinical *single case causality*. The article outlines models of medical professionalism such as *technical rationality* and *practice epistemology*, and characterizes features of professional expertise such as *tacit knowledge*, *reflection in action*, and *gestalt cognition*.

**Conclusions:**

Consequences of a methodological and logistical advancement of clinical judgment are discussed, both in regard to medical progress and to the renewel of the cognitive basis of the medical profession.

## Introduction

The medical profession is ‘a vocation in which a doctor's knowledge, clinical skills, and judgement are put in the service of protecting and restoring human well-being’[1]. A basis of this profession is *clinical judgement*. It lies at the heart of the doctor's connoisseurship, expertise and skills, being ‘almost as important as the technical ability to carry out the procedure itself’[2]. Clinical judgement is developed through practice, experience, knowledge and continuous critical analysis. It extends into all medical areas: diagnosis, therapy, communication and decision making. However, there are also other views on clinical judgment which disregard it as notoriously fallacious, as an unfathomable and irrational black box: ‘a smokescreen for not having read this week's *NEJM* or *Lancet*’[3–5].

During recent years, there has been a newly arising interest in this topic, and there has been an array of investigations on clinical judgement. They were, however, mainly restricted to the role of clinical judgement in communication, diagnosis and decision making [6–10], not taking into consideration its capacities for therapy assessment: its potential competence and validity, its susceptibility to error and bias, and the question of whether it could possibly be optimized and professionalized. The present article enters into this issue and highlights the interrelations of clinical judgement, therapy assessment, and medical professionalism.

## Clinical judgement and positivistic therapy assessment

The reputation of clinical judgement underwent substantial transformation during the last century. Initially, the clinically skilled and scientifically competent doctors and their judgements were the main impetus for treatment decision, therapy assessment and medical progress. With the rise of modern research methodology, however, the fallacious aspects of clinical judgement were increasingly emphasized. It was presumed that personal judgement would be unable to go beyond a simple *post hoc ergo propter hoc*, and could at best accomplish something like simple, intuitive, low-quality correlational statistics [11,12]. A primary mission therefore became ‘to guard against any use of judgement’[13], and it was executed through clinical trials. Yet this general discredit of personal judgement was not based on systematic investigations, but on anecdotal examples of naivety and error and on the general low esteem of personal cognition in the times of neopositivist [14] and fallibilist [15] epistemologies.

When judging a treatment outcome, one is basically confronted with the causality question of whether the outcome is causally connected to the treatment manoeuvre. For the assessment of such therapeutic causality, the randomized controlled trial (RCT) has become the gold standard. The RCT incorporates paradigms of famous philosophers and methodologists who claimed causality assessment to be valid and reliable only on the basis of: *experiment* (Francis Bacon, 17th Century) [16], *many repeated observations* (David Hume, 18th Century) [17], *comparison* (John S. Mill, 19th Century) [18], *randomization* (Ronald Fisher, 20th century) [19]. Today's medical methodology is outlined accordingly: the assessment of therapeutic causality is claimed to require an interventional study (=experiment) of a cohort (=many repeated observations), with a control group (=comparison), and with random allocation of patients to the intervention or the control group (=randomization).

Particularly important in the canon of these paradigms is Hume's seminal position that causality assessment requires repeated observations, and is not possible in single cases. Consequently, even when randomization cannot be set up, clinical epidemiology asks at least for a group comparison [20,21].

Accordingly, if an individual doctor wants to judge a therapeutic effect, he/she will have to treat many patients with the respective therapy and treat many other patients with a different (or no) therapy and gain a sense of the average outcomes in both groups as well as of their difference – while taking into account spontaneous variations and effects of prognostic factors, adjunctive therapies, context factors, etc. This kind of judgement is obviously beyond the capacity of the singular practitioner. Not surprisingly, the classic article on evidence-based medicine [22] restricts *clinical expertise* to diagnosis and to identification of the patient's perspective, and does not include judgement about the effects of care. Concerning therapeutic effectiveness, the doctor seems to be totally dependent on external evidence. Seen from this perspective, reliable personal judgement, experience and expertise appear to be a self-delusion.

## Technical Rationality – epistemology of practice

The corresponding model of professionalism is *Technical Rationality* (the Positivist epistemology of practice), conceiving intelligent practice as *application* of external science: the practitioner hands his problem over to the external scientist, who, after having solved the problem, returns scientific knowledge and scientific directions to practice [23]. Indeed, many modern therapies come from the realm of external science: developed by pharmaceutical industries, tested by epidemiologists and statisticians, licensed by agencies, assessed in reviews and Health Technology Assessment-reports, directed through guidelines. For the practicing doctor, all which remains is to clarify whether his/her patient matches with the inclusion criteria of the respective ‘best evidence study’, and whether the therapy matches his/her patient's life perspectives. The doctors themself are needed only for the transmission of the scientific results to the singular patient, with little necessity for their own clinical judgement.

What are the consequences? The model of Technical Rationality shifts emphasis from the patient–doctor relationship, centred on individual help, to the client–provider contract, centred on the application of certified health technologies. The practical realization of this model, together with economic restrictions and liability issues, leads to extensive external regulation of the doctor's activities, to diminishing autonomy, deprofessionalization, proletarianization and over-management of medicine. Consequences are an overwhelming bureaucratic work load, restricted expert performance, and minimized time for the individual patient [23,24]. In many countries, doctors feel utterly demoralized by their situation [25].

Still, in contrast to the positivists' theory and ideal of Technical Rationality, the practice of the medical profession has often been described as a combination of *practical science* and *artistry*, with its own characteristic features, distinct from those of natural or theoretical sciences [23,24,26,27]. The model of Technical Rationality has been shown to be grossly oversimplified and applicable only to simple, repetitive and novices' situations, but not to the complexity which generally characterizes real-life professionalism [23,26]. For a more elaborate epistemology of professional practice several further traits, in addition to external knowledge, are characteristic –*tacit knowledge*, *reflection in action* and *Gestalt cognition*[23,26,28–30].

*Tacit knowledge* (implicit knowledge) generally constitutes much of expert knowledge. Experienced and competent professionals do not only rely on explicit factual knowledge but also on tacit knowledge [23,26,28,31,32]. The competent practitioner ‘makes innumerable judgements of quality for which he cannot state adequate criteria, and he displays skills for which he cannot state the rules and procedures. . . . he is dependent on tacit recognitions, judgements, and skillful performances.’[23] Practitioners know more than they can say, and it is the tacit knowledge which distinguishes the master from the apprentice. Most obviously, tacit knowledge is present in outstanding musicians, athletes or chess players. Tacit knowledge shows a higher correlation to professional success than conventional intelligence [26,29]. When experts try to follow the demand for rationalization, and to find elements and rules for their performance, they easily regress to the level of a novice [28,29,33,34]. Experts' knowledge with its striking flexibility, sensitivity to context and individual orientation cannot be replaced by formalization [26]. This also applies to scientific discovery: the abilities to see a problem, to anticipate the unknown, and to find a new route to insight and knowledge are all based on tacit dimensions [30].

*Reflection in action* becomes relevant when the practitioner deals with a situation of uncertainty, instability, uniqueness and conflicting values. When confronted with problems for which explicit guidelines or implicit knowledge are insufficient, or when spontaneous performances yield unexpected results, the practitioners can become researchers in their own practice. They enter into a reflective conversation with the situation in order to a find solution. Open for the discovery of new phenomena, they become artistic and creative, and can eventually produce new insight and knowledge. Through reflection, they can also surface and criticize the tacit understandings that have evolved around repetitive observations and around guidelines. In this way, reflection can serve as a corrective to overlearning [23,26].

*Gestalt cognition* assesses the wholeness of a pattern (or a substance, or a meaning) that is irreducible to its parts and conceivable independent from its particulars [35–37]. While stochastic approaches assess correlations, Gestalt-oriented approaches assess patterns. Personal experience can transform into *Gestalt* cognition, which can be recast into the logic of tacit thought, and can eventually be manifested even in the tacit power of the scientific or artistic genius [30]. It is this capacity for *Gestalt* cognition which enables the expert's connoisseurship, that is, his exceptional ability to swiftly interpret situations and to exhibit outstanding performances [26,29,38]. *Gestalt* cognition also promotes the capacities for reflection in action.

An important question now is whether these features of professionalism are of any relevance to the judgement of therapy effects. The answer must be definitely negative, that is, as long as David Hume's dictum is held to be true: as long as single case causality assessment is considered as principally impossible. Yet, the school of *Gestalt theory*[39], and also other epistemologists [40–42], raised opposition against Hume's doctrine and pointed at novel ways to causality recognition which are also relevant for medicine.

## Gestalt cognition in the judgement of therapy effects

Karl Duncker, in an essay on *productive thinking*[43], demonstrated with everyday examples that certain cause–effect relations can be assessed in single case situations – when the Gestalt (feature, pattern, substance) of the cause stretches into the Gestalt of the effect and can be perceived there. Simple examples are: the wetness of the rain causes the wetness of the street; the sequence of the trumpet sounds cause the rhythm and melody of the echo. Both examples offer certainty about the causal relation even in the mere singular case: the rain example, because the substance of the cause (wetness, water) continues into, or is identical with, the substance of the effect; and the trumpet example, because the pattern of the trumpet sounds (rhythm, melody) is actively triggered by the trumpeter himself and reappears in the pattern of the echo.

Altogether there are three levels of valid causality assessment: [44] (1) When, as in the rain example, a *Gestalt unity* of cause and effect can be apprehended, one can be certain about the cause–effect relation even without experimental activity. (2) When, as in the trumpet example, there is no unity but a *Gestalt correspondence* between cause and effect, one can also be certain about causality, but only when the cause is triggered through intentional activity. (3) When there is neither a Gestalt unity nor a Gestalt correspondence, one needs intentional activity in two instances: in triggering the cause *and* in controlling the conditions under which the effect is being observed, that is, one needs to do a controlled trial, an RCT.

In medicine, indeed, one can find many examples not only for level 3, but also for level 1 and 2 assessment: *Gestalt unities*, for example, when an implanted prosthesis becomes a new wall of a ruptured aorta and thus prevents further extravasation [45]; and *Gestalt correspondences*, when one has examples such as the following ones. *Time-correspondence*: when an uncontrollable postpartum bleeding because of *placenta accreta* ceases immediately after vasopressin infiltration [46]. *Time-pattern-correspondence*: when a persistent hiccup stops on day 8 exactly when the patient smokes marijuana, recurs on day 9, and again disappears (persistently) on day 10 right after marijuana is being smoked once more [47]. (The design of conventional N-of-1-studies follows such time patterns.) *Space-pattern-correspondence*: when 24 hours after intracutaneous injections of Botulinum toxin at ten different sites of a chronically hyperhidrotic palm, there were corresponding anhidrotic areas growing exactly around the ten injection sites, finally flowing together and thus creating a persistent total anhidrosis [48]. *Morphological-correspondence*: when conduct anaesthesia creates an anaesthetized area corresponding with the innervation area of the blocked nerve; or when external fibers of the nerve are blocked first and when correspondingly the onset of anaesthesia starts proximal, spreading to distant areas only later on [49]. *Dose–effect-correspondence*: when catatonia ratings in a woman with schizoaffective disorder improve in direct correspondence with zolpidem plasma concentrations [50]. *Dialogual-correspondence*: when a 5-year-old autistic boy, who had never spoken a word but only screamed chaotically all his life, is brought to interactive Nordoff–Robbins music therapy, and when he increasingly echoes a variety of musical elements presented by the piano therapist, until he also echoes sung words and thus develops a growing vocabulary [51]. *Parallel-test-result-correspondence*: when the infection in a woman's swollen and deeply blue finger, having been bitten by a swan, was first unsuccessfully treated with oral cephradine, and then, after the wound swab culture had demonstrated a *Pseudomonas aeruginosa* resistant against cephradine but sensitive to ciprofloxacin, was treated with ciprofloxacin and was rapidly healed [52]. *Complex-prediction-and-observation-correspondence*: when chronic anal fissures with sustained internal sphincter hypertonia [53] and subsequent reduced perfusion of the posterior midline anoderm [54] are interpreted as ischemic ulcers [55], and when therefore the external application of isosorbide dinitrate is expected to provoke a sequential process as follows: first a reduction of anal pressure, second an increased perfusion, third a reduction of fissure related pain, fourth a healing of fissure – and when exactly this sequence is observed [56].

Clinical practice is full of such cases, partly trivial, partly exotic. Their common feature is not eminent effect size, but the Gestalt-based approach to therapeutic causality. Such cases do not only deal with short term effects, but also with their long-term continuations [44]. Such cases demonstrate that – in principle, when Gestalt-criteria are accessible – clinical judgement can reach beyond simple *post hoc ergo propter hoc*. Combinations of different Gestalt-elements even allow subtle and sophisticated judgements, and through integrating repeated case judgements one can build up valid experience.

Based on single case causality assessment one can produce *internal* evidence (personal judgement, experience, expertise) as well as *external* evidence (case reports and case series in which the respective Gestalt criteria are explicitly taken into account). The explication of the Gestalt-criteria raises this kind of causality assessment above mere implicitness, and extricates the case reports and case series from their traditional low evidence level.

Of course, protection against error must be given consideration. As is necessary in all fields of enquiry, it is important to observe and exercise the following criteria: self-critical attitude; comprehensive and clear observation; assemblage of the essential details; analysis of well-known factors of misperception [57]; reflective explication of the judgement criteria; and, as far as possible, communication with colleagues, or a publication and a critical appraisal by the medical community.

Notably, the individual clinician can replicate or revise the therapeutic observations of his/her fellow colleagues, whereas RCT results – which he/she can never check in hi/hers non-randomized routine patient care – have to be followed blindly.

## Two approaches

The three levels of causality recognition (see above) imply two different kinds of approaches to therapy assessment: one based on cohort comparison and ultimately demanding RCT technology; and one based on case analysis and ultimately requiring Gestalt criteria. The first approach can assess, on cohort level, the superiority of one treatment compared to another but cannot evaluate treatment effects in individual patients. The second approach can assess individual effectiveness but cannot determine general superiority.

The first approach – with the RCT as gold standard – is currently given priority in clinical research. RCTs make essential contributions to patient care and constitute an indispensable feature of medical professionalism. Nevertheless, the RCT has its limitations: it cannot be conducted by an individual doctor who wants to investigate therapeutic procedures on his own initiative; impediments are the logistic and bureaucratic complexity, extensive regulations, and the excessive costs that require a powerful financial backing [58]. An RCT can only be conducted under restricted circumstances: when the respective disease is sufficiently frequent, so that enough patients can be recruitedt [59,60]; when neither the test or control therapy is strongly preferred [61]; and when both therapies seem to be similarly successful (equipoise) [62]. The RCTs usually do not assess treatments in the daily clinical routine situation [60,63] (but often evaluate substantially different modalities of intervention, also co-interventions, treatment setting, length of treatment and follow-up, patients characteristics, diagnostic procedures, outcome measure, etc [60,63,64]. Furthermore, RCTs seem inappropriate for complex and skill-dependent treatments and can systematically distort their evaluation [65], and finally, the cost of the studies (average costs per RCT with public health relevance are estimated at 12 million US dollars [66]) leads to a commercial bias by privileging patentable treatments from the pharmaceutical industry for large patient populations. Disadvantaged, on the other hand, are non-profit or low-profit treatments, non-patentable drugs, and non-pharmacological treatments like surgical procedures, educational and lifestyle interventions, diet, weight-loss programmes, exercise, physiotherapy, sanitation, treatments for neglected diseases, for children, for maternal health, antidotes for poisoning, rare diseases, etc.

The second approach, with Gestalt-oriented therapeutic causality assessment, also has specific limitations. It is only applicable when Gestalt criteria are accessible. How often this occurs in clinical practice is not known at present. A vague orientation might be supplied by a retrospective analysis of 122 consecutive general practice interventions. A total of 82 treatment cases were ‘evidence based’ but, quite remarkable, 51 of these were based on ‘convincing *non*-experimental evidence’[67]. As a consequence, the authors of this analysis demanded ‘an appropriate paradigm of evidence based practice rather than that determined solely by clinical trials’: ‘We believe that for general practice, and possibly in other settings too, the most important evidence may be found in developing alternative methodologies which complement conclusions from randomized controlled trials.’ Gestalt-based therapy assessment might be one of these methodologies.

The primary importance of Gestalt-oriented assessments lies in clinical situations in which the question of individual effectiveness is predominant: when clinicians are gaining new insights in their practice (‘reflection in action’); when individual tailoring of treatment is necessary (e.g. pain treatment, complex interventions, certain surgical procedures, physiotherapy); when positively RCT-tested treatments are absent, or insufficient, or without benefit for the individual patient (for positive RCT results the ‘number needed to treat’ usually lies between 2 and 100, i.e. 50–99% of patients do not benefit from the respective treatment [68]); when responders and non-responders have to be identified; or when unexpected potential side effects are observed. In clinical practice, the individual effectiveness of a treatment for the particular patient is more important than the (usually small [59]) superiority on cohort level, which is the predominant question of a comparative trial. The more skill-based a therapy – that is, the more the therapy is an inherent part of treatment actions that depend on personal abilities – the less relevant it is to know the comparative efficacy *as such*, and the more important the matters of clinical judgement can become.

An open question, both for RCTs and case studies, is the topic of generalization. Unless the RCT samples and the case patients are randomly selected from the target population (which they almost never are) and unless all treated patients are responders (number needed to treat = 1) or response criteria are fully transparent, there will always be substantial indeterminacy as far as generalization is concerned [68]. Generalization will seem more legitimate when based on cohorts than only on cases. However, a series of consecutive cases is also a cohort, and it already includes replications.

## Medical innovation

Clinical reasearch has lead to a substantial improvement of medicine. Still, a renaissance of clincial judgement – critically based on gestalt criteria – could offer a methodological expansion for the field of professional therapy assessment. Not only clinical practice could benefit from it, but also clinical research and medical innovation. After the golden ages between 1930 and 1965, when seminal discoveries irrevocably changed medicine, the rate of remedial innovation has continuously declined despite billions of dollars invested [69]. The great discoveries were made by ingenious pioneering individuals who combined basic science and passionate clinical work: driven by the desire to cure patients, guided by scientific thinking; open to the unexpected, and little hampered by bureaucracy and costs. For their discoveries, clinical judgement was indispensable. Relying on small sample sizes and discarding therapies unless there were obvious effects in 10–20 patients, they found spectacular results and thus expedited innovation. Effects that could only be seen in large trials were regarded as not worth bothering about. The spectacular, ground-breaking discoveries such as sulphonamids, penicillin, cephalosporins, neuroleptics, antidepressants, steroids, etc. would possibly not have been made within the requirements of modern research bureaucracy [59,70].

Since then, the increase of administrative burden, negative attitudes towards the innovative clinician, and astronomical research costs forced large sections of treatment development to shift from the patient-oriented doctor to patent-oriented industry [69,70]. Given the present conditions, future therapy developments are unlikely without substantial industrial support, particularly without patent protection. However, industrialized drug development is regarded as inefficient: dominated by marketing research, mass procedures, and by techniques like screening, computerized drug design, and genomics – instead of putting most emphasis on the creative, ingenious individuals with broad knowledge of medicine and biology [71,72]. The primary goal is large sales, and the bizarre culmination of this goal is to design drugs for healthy people that ‘sell to everyone’[73]. For most diseases, however, therapeutic progress is pathetic. The less lucrative a disease, the more is it neglected.

The other side of the same coin is visible in clinical medicine where the spirit of innovation has been exorcised. Rigid regulations, endless documentation and rapid throughput of patients allow little time for the contemplation of clinical problems. Clinical thinking and clinical judgement have come into seeming conflict with efficiency and economics. Discoveries and innovations by clinicians (‘the spirited pursuit of the unknown – so long a defining quality of medicine’[74]) are being discouraged and have fallen into disrepute. The brightest and most imaginative individuals, once attracted by clinical medicine, head to other disciplines, while those who stay risk becoming stultified by repetitious routine. A tragedy, because ‘clinical champions’ would have the skills and scientific background for relevant observations and new medical insights, would be able to identify critical questions and seek satisfying solutions [74].

## Consequences for the medical profession

Traditional medical methodology does not sufficiently reflect the necessities and possibilities of individual effectiveness assessment. As a consequence, clinical judgement is considered the non-objectifiable part of medicine, allowed to pursue a merciful existence ‘in the darkness of the doctor-patient relationship’[75]. Similarly, case reports and case series, though on the other hand often designated as ‘cornerstones of medical progress’[76], are considered as the lowest level of evidence: ‘the least-publishable unit in medical literature’[77]. Thus, in the shadow of these preconditions and premises, individual clinical judgement and clinical expertise have remained a blind spot in modern medicine.

Investigating the principles and criteria of individual causality assessment could shed some light into this darkness. When taking notice of the full scope of causality assessment – not only of the RCT-dominated level 3, but also of the Gestalt-oriented approaches of level 1 and 2 – one could bridge the two otherwise incommensurable worlds of medical professionalism: the realm of technical rationality that is based on external evidence; and the realm of practice-innate rationality that is born out of observation, reasoning, tacit knowledge and reflection in action.

During the second half of the 20th Century, medical professionalism benefited from the evolution of *external* evidence, with the RCT as gold standard. A consequential new step would be to additionally evolve the methodology of clinical judgement, and the potentials for *internal* evidence. To this purpose, the Gestalt-oriented methodology of individual effectiveness assessment has to be further investigated, the criteria of valid judgement have to be established, and a methodically reshaped clinical judgement has to be newly implemented into all levels of medicine: into individual therapy situations, daily communication, journal publications, and general health care decisions. Moreover, in medical education, not only statistics should be taught, but also the epistemology of clinical judgement.

It is also necessary to reverse the exodus of research-oriented clinicians and clinically oriented basic scientists [69]. Novelty and originality, imagination and inquisitive thinking – all this needs to be cultivated, not penalized. More advantage should be taken of the experiential knowledge of clinicians. Dealing with suffering patients every day, making important observations regarding illness, interventions and patients needs, the clinicians can observe practical consequences of therapeutic applications and of health care directions given to them by the knowledge-creating institutions. ‘In caring for patients, clinicians constantly perform experiments. During a single week of active practice, a busy clinician conducts more experiments than most of his laboratory colleagues do in a year.’[78] These observations, however, have little influence backwards on the more general levels. To take advantage of this resource, methods and logistics should be developed for skimming off the knowledge pool that is build up through the clinicians' daily experience. Systems for accessing high-quality judgements need to be created.

The clinicians should again be enabled to participate autonomously in clinical research. Institutional and financial barriers should be reduced, and many more small trials should be conducted, particularly on concepts and products that are not supported by the pharmaceutical industry. Being unafraid of small studies looking for large effects, rather than conducting large trials looking for small effects, might well accelerate medical progress [69]. Furthermore, analysing and publishing clinical judgements of highly skilled and ingenious clinicians could profoundly contribute to medical progress and education.

One may also have to introduce something that actually ought to be considered a matter of self-necessity, and is still vanishing from the present clinical agenda – a culture of following up the patients who have been treated in the daily practice. Judging the result of the individual patient's treatment as thoroughly and comprehensively as possible, and whether there is sufficient cure or relief, should be part of routine practice. The resulting increase in consultation fees might well be counterbalanced by reduced costs because of better outcome and less doctor-hopping by the patients.

The medical profession would profit from a further evolution of the methods and strategies of clinical judgement. As long as the doctors are, in principle, considered incapable of judging whether they help their individual patient or not, they will need strict external guidance. Clinical judgement, and its further development, is therefore a key issue for the future destiny of the medical profession. An advancement of clinical judgement could grant a renewed cognitive basis for medical expertise and medical professionalism, and could increase both the intellectual and practical autonomy of the doctor.
